# Influence of hormonal status in hereditary hemorrhagic telangiectasia – analysis of an online patient questionnaire

**DOI:** 10.1186/s12884-026-08928-2

**Published:** 2026-03-20

**Authors:** Freya Droege, Kruthika Thangavelu, Angela Koeninger, Eva Maria Huessler, Stephan Lang, Boris A. Stuck, Urban W. Geisthoff

**Affiliations:** 1 Department of Otorhinolaryngology, Head and Neck Surgery, Essen University Hospital, and VASCERN HHT Reference Center, University Duisburg-Essen, Hufelandstrasse 55, Essen, 45122 Germany; 2 Department of Otorhinolaryngology, Head and Neck Surgery, and VASCERN HHT Reference Center, University Hospital Marburg, Philipps-Universität Marburg, Baldingerstrasse, Marburg, 35043 Germany; 3 Clinic St. Hedwig of the Order of St. John, Department of Gynecology and Obstetrics, University of Regensburg, Steinmetzstrasse 1-3, Regensburg, 93049 Germany; 4 Institute for Medical Informatics, Biometry and Epidemiology, Essen University Hospital, University Duisburg-Essen, Hufelandstrasse 55, Essen, 45122 Germany

**Keywords:** Hereditary hemorrhagic telangiectasia (HHT), Hormonal status, Contraceptives, Pregnancy, Hormone intake

## Abstract

**Background:**

Hereditary Hemorrhagic Telangiectasia (HHT) is a rare vascular disorder with mucocutaneous telangiectasia. Hormones are hypothesized to play a role in reducing bleeding in these patients; however, clear evidence is currently lacking. The aim of this study was to further investigate this hypothesis by assessing the impact of altered hormonal status on HHT severity.

**Methods:**

This cross-sectional electronic questionnaire, available in English and German, was distributed with the support of various self-help groups. It included sections on the general medical history of HHT and the influence of hormones on disease symptoms. Data were collected between March 2016 and August 2017.

**Results:**

Of the 477 respondents with HHT, 326 were female (68%). With increasing age, female patients experienced more severe epistaxis compared to male patients, with a mean age of 54 years (standard deviation ± 11.5 years, range [28,83]). Of the 228 female patients who reported having taken hormones, 73% used them for contraception and 57% for hyper- or dysmenorrhea. Regarding hormone intake, 52% of the patients taking combined estrogen and progestin therapy experienced less epistaxis. Most female patients stated an increase in epistaxis during puberty (41%) and pregnancy (39%), predominantly in the second trimester.

**Conclusions:**

In HHT, variation in levels of hormone concentrations seem to contribute to symptom severity. Off-label use of hormonal products in women with HHT may be considered.

**Trial registration:**

Clinicaltrials.gov: NCT02690246, Registration Date: 2016–02-09.

**Supplementary Information:**

The online version contains supplementary material available at 10.1186/s12884-026-08928-2.

## Introduction

Hereditary hemorrhagic telangiectasia (HHT), also known as Osler-Weber-Rendu syndrome, is a rare autosomal dominant disease with a prevalence of 1 in 5000–8000 [1, 2]. It is caused by pathogenic variants in the genes attributed to transforming growth factor β (TGFβ) pathway [3]. Patients with HHT typically develop vascular dysplasia of the skin and mucosa. Visceral malformations are most commonly observed in the hepatic, pulmonary, and cerebral vascular systems. More than 90% of the affected individuals present with spontaneous recurrent epistaxis [4–7].

Mucocutaneous telangiectasia and epistaxis (= nosebleeds) are the most common presenting symptoms. The latter also happens to severely affect the quality of life in these individuals and is also proven difficult to control or treat. Numerous different preventive and acute treatment options exist, such as topical nasal medications, nasal packing, or surgical options such as laser therapy or complete closure of the nostrils [5, 8–10]. Research on medical therapy of epistaxis has been performed, with differing therapies shown to have variable success [11, 12].

The potential role of hormones in reducing epistaxis in patients with HHT has been suggested since the 1950s. It has served as a rationale for the use of hormonal therapy in the treatment of epistaxis and bleeding from vascular malformations. However, clear evidence that epistaxis severity in HHT varies in response to changing hormonal levels remains lacking [12, 13]. The aim of the present study was to further investigate this concept by assessing the impact of altered hormonal status on disease severity in women across different age groups and comparing these findings with those in men.

## Materials and methods

### Patient recruitment

An electronic questionnaire in English and German was administered online via the Survey Monkey® platform (see Additional File 1). Survey links were distributed through various patient self-help groups (see acknowledgements). The online questionnaire was designed to prevent participant tracking, thereby ensuring complete anonymity. The survey was initially developed by the authors in German subsequently translated into English (see acknowledgements). Participants were able to choose between the two language versions. Participation in the study was possible between March 2016 and August 2017. The diagnosis of HHT was established according to the previously published criteria [14] (see also Additional File 2).

The study was conducted in accordance with the Declaration of Helsinki and was approved by the Ethics Committee of the University Duisburg-Essen (15–6429-BO, date of approval: 19th November 2015). Prior to participation, patients were provided with written information about the study, including contact details of the first author for additional inquiries. Completion of the online questionnaire constituted provision of written informed consent by all participants (see online questionnaire in the Additional File 1). The study was registered at www.clinicaltrials.gov (NCT02690246).

### Survey description

The questionnaire included sections about the general medical history of HHT (including questions regarding the Curaçao criteria [15] for reassuring the HHT diagnosis) and patients’ epistaxis severity measured with the Epistaxis Severity Score (ESS, the ESS can reach values between 1 and 10, where a larger value represents a more severe epistaxis [16]; questions number 4–48 in the Additional File 1). Participating women were asked to report variations in disease severity during different hormonal phases of their life, including puberty, menstruation, pregnancy, the postpartum, and post menopause. In addition, information on hormone intake and previous gynecological interventions was collected. Women were also asked to categorize their menstrual bleeding relative to women without HHT (questions number 100–118 in the Additional File 1).

### Statistical analysis

Descriptive statistics were done for hormonal changes affecting epistaxis severity, and various patient groups were compared using the relative risk (RR) for binary outcomes and the difference in means (Diff) for continuous outcomes. Linear regression was used for associations between age, male or female sex and ESS. To compare the change in symptoms (more symptoms = + 1, no change in symptoms = 0, less symptoms = −1) in different life times between patients with HHT type 1 and patients with HHT type 2, mean differences were calculated with 95% confidence intervals (CI) and p-values using a two-sided t-test. The means itself with the 95% CI were calculated using a one-sided t-test. Contingency tables were analyzed using a chi-square test of independence (null hypothesis), if at least 80% of the cells had expected cell counts of at least 5. All analyses were performed using Stata 14.0, SAS software 9.4 (Statistical Analysis System, SAS Institute Inc., North Carolina, USA) and IBM SPSS statistics version 27.

## Results

### Characteristics of the patient cohort

A total of 588 patients from the 915 survey respondents (64%) had a definite diagnosis of HHT, 15 participants (2%) had a possible diagnosis of HHT and the rest (34%) did not have a diagnosis of HHT based on the Curaçao criteria (see Additional File 2).

Further data and analyses refer to the 588 patients with confirmed diagnosis of HHT. Notably, not all of the 588 patients with confirmed HHT completed every section of the questionnaire. Thus, the reported numbers and percentages pertain only to the cohorts that fully completed each set of questions. Sex was answered by 477 patients (= 81%). Out of these 477 respondents about two-thirds of the respondents were female (proportion of female respondents: 326/477 = 68%; proportion of male respondents: 151/477 = 32%). The mean age of all respondents was 56 years (standard deviation (SD): ± 12.2 years, range [20, 83], *N* = 150), with females being younger than males. 161 participants (proportion of respondents: 161/588 = 27%) fulfilled the German version of the questionnaire. Percentages pertain only to the cohorts that fully completed each set of questions; i.e.: results are analyzed in different subsets, depending on the questions answered by the patients. In 321 patients of 578 respondents (= 56%), genetic testing was performed with most patients having been diagnosed with HHT type 2. The patients with HHT reported that the first symptoms occurred at a mean age of 18 years (± SD of 14.0 years, range: [0, 67], *N* = 507). Most patients suffered from epistaxis and telangiectasia and more than every third patient had a gastrointestinal involvement (Table 1).

**Table 1:** Patient characteristics for all participants and for the participants divided by sex

	**All** (*N* = 588)		**Females** (*N* = 326)		**Males** (*N* = 151)	
		**NA**		**NA**		**NA**
Age in years mean ± SD [range]	56 ± 12.2 [20,83]	438	54 ± 11.5 [28, 83]	222	60 ± 12.7 [20,81]	105
Age at disease onset in years mean ± SD [range]	18 ± 14.0 [0, 67]	81	18 ± 14.1 [0, 66]	53	18 ± 13.6 [0, 66]	12
Genetic testing N (%)	321 (56)	10	180 (56)	4	82 (56)	4
HHT type 1	89 (28)		54 (30)		17 (21)	
HHT type 2	132 (41)		76 (42)		38 (46)	
SMAD 4	3 (1)		1 (0.5)		2 (2)	
Epistaxis N (%)	532 (97)	37	309 (95)	1	146 (98)	2
Telangiectasia N (%)	557 (96)	10	309 (95)	1	146 (97)	1
Visceral lesions (any) N (%)	399 (68)	189	236 (72)	90	96 (64)	55
Gastrointestinal bleedings	191 (36)	50	108 (35)	16	55 (39)	9
CVM	73 (14)	50	42 (14)	17	19 (13)	9
PAVM	251 (46)	37	146 (46)	8	61 (43)	8
HVM	135 (26)	59	79 (26)	20	26 (19)	11

*N* number of participants with specific condition, *NA* Number of missing values, *CVM* cerebral vascular malformation, *PAVM* pulmonary arteriovenous malformation, *HVM* hepatic vascular malformation

### Differences in male and female patients

**Fig. 1:**
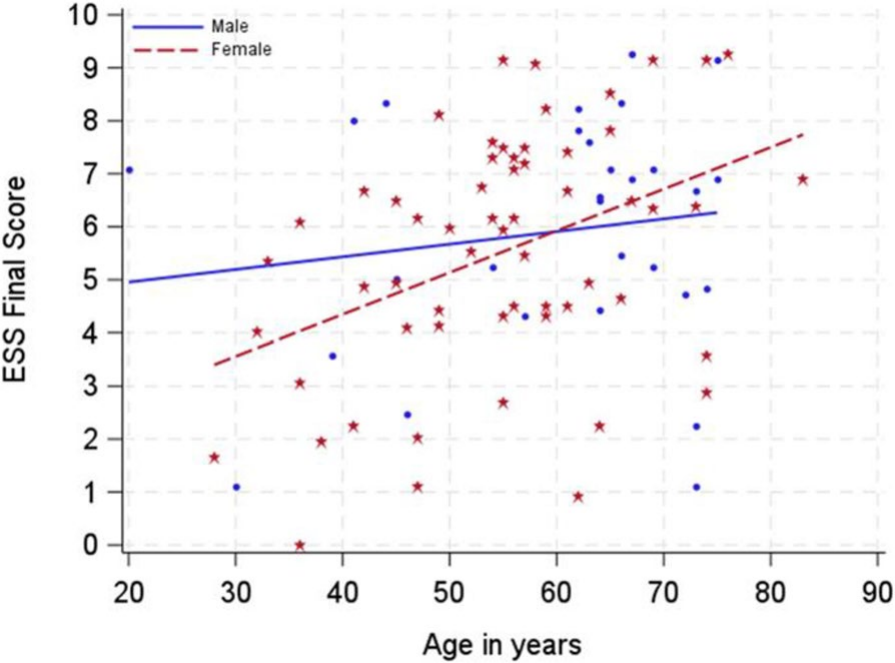
Scatter plot of ESS final score versus age in years with estimated regression lines for male and female HHT patients. Older female patients suffered from more epistaxis compared to male patients. The estimated epistaxis severity score (ESS) is estimated to be −3.28 (95% CI [−7.70, 1.13]) lower in women than in men at a hypothetical age of 0 years. The increase of the ESS per year is estimated to be 0.024 (95% CI [−0.033, 0.081]). The additional increase of the ESS per year in females compared to men is 0.055 (95% CI [−0.019, 0.129]). The number of patients for this analysis was 88. CI = confidence interval, HHT = hereditary hemorrhagic telangiectasia

Epistaxis severity did not differ significantly between male and female patients (ESS: females [mean ± standard deviation (m ± SD)]: 5.7 ± 2.2, range [1, 10], *N* = 195; males [m ± SD]: 6.1 ± 2.2, range [1, 10], *N* = 88; Diff: −0.4; 95% CI [−0.95, 0.15]). In line with this, most women and men suffered from anemia (proportion of female respondents suffering from anemia: 233/291 = 80%; proportion of male respondents suffering from anemia: 111/135 = 82%, relative risk for women compared to men (RR for women): 0.97, 95% CI [0.88, 1.07]). Although men showed a trend toward requiring more blood transfusions, the difference was not statistically significant (proportion of females needing transfusions: 89/246 = 36%; proportion of males needing transfusions: 47/106 = 44%; RR for women: 0.82, 95% CI [0.62, 1.06]). There was no significant difference in average hemoglobin level between women and men (females: [m ± SD]: 10.6 ± 2.5 g/dl; range [3.0, 17.6], *N* = 190; males [m ± SD]: 11.0 ± 2.7 g/dl; range [3.9, 17.5], *N* = 100; Diff: −0.4; 95% CI [−1.02, 0.22]). No difference in rates of iron intake between the sexes could be observed (proportion of female respondents: 271/325 = 83%; proportion of male respondents: 125/149, 84%; RR for women: 0.99, 95% CI [0.91, 1.08]), while the distribution of the route of administration seems to differ between the sexes (*p* = 0.024; Table 2). With increasing age, females were estimated to experience more severe epistaxis compared to male patients, whereas the ESS was lower in women than in men at younger ages. The additional increase of the ESS per year in women compared to men was estimated to be 0.055 (95% CI [−0.019, 0.129]; *N* = 88; Fig. 1). This means that after 20 years of aging, the score is estimated to increase 1.1 points more in women than in men. A male patient of 20 years with an ESS of more than 7 was regarded as a potential outlier, distorting the analysis. Therefore, the analysis was repeated without the patient data thought to be an outlier (Fig. 1). The additional increase in ESS in women compared to men yearly was still estimated to be 0.034 (95% CI [−0.049, 0.116]). Thus after 20 years, women were estimated to have an ESS with an additional 0.7 points compared to men (the minimal important difference of the ESS is 0.71[17]).

**Table 2:** Iron supplementation in patients with HHT

		**All** (*N* = 588, NA = 11)		**Females** (*N* = 326, NA = 1)		**Males** (*N* = 151, NA = 2)		**RR** [95% CI], **Chi-Square test p-value**
			**N**		**N**		**N**	
Iron intake	N (%)	480 (83)	577	271 (83)	325	125 (84)	149	0.99 [0.91, 1.08]
	Only oral intake	258 (54)	480	157 (58)	271	60 (48)	125	*p* = 0.024
	Only intravenous intake	47 (10)	480	30 (11)	271	9 (7)	125	
	Oral and intravenous intake	175 (36)	480	84 (31)	271	56 (45)	125	

*N* number of participants with specific condition, *NA* Number of missing values

### Hormone intake and contraceptives

At least one question about their hormonal status or intake was answered by 352 patients (proportion of respondents: 352/588 = 60%). Most patients were female (proportion of female respondents: 316/352 = 90%; proportion of male respondents: 30/352 = 9%, sex unknown: proportion of respondents: 6/352 = 2%).

Some form of hormonal intake was reported in 242 patients (proportion of respondents: 242/347 = 70%). Although questions about the hormonal intake and effect were directed to female patients, 8 male patients also stated hormonal intake (in 236 cases the sex was known (proportion of respondents: 236/242 = 98%); proportion of respondents: female 228/236, 97%, proportion of male respondents: 8/236 = 3%). In these eight men, all reported a systemic intake (100%) and the pharmaceutical ingredient was known in 38% (tamoxifen: 3 of 8 respondents = 38%). In 4 cases (proportion of respondents: 4/8 = 50%) the reason for the hormone intake was stated: bleeding control (proportion of respondents: ¾ = 75%) and osteoporosis (proportion of respondents: 1/4 = 25%).

The following results represent data only from female respondents.

The largest group of female patients took estrogen- and/or progestin-preparations (106/120 = 88%, (estrogen: 32/106 = 30%, progestin: 18/106 = 17%, combined estrogen and progestin: 56/106 = 53%). Other active pharmaceutical ingredients such as testosterone (4/120 = 3%) and antiestrogens (proportion of respondents: 10/120 = 8%, (selective estrogen receptor modulators (SERM): 8/10 = 80% (tamoxifen: 6/8 = 75%, raloxifene: 2/8 = 25%), aromatase inhibitor: 2/10 = 20%)) were also reported (multiple answers were possible). Most of the female patients stated that they took hormones orally (proportion of respondents: 205/219 = 94%). Hormonal patches were reported by 12 females (proportion of respondents: 12/219 = 5%) and 10 patients used hormonal creams (proportion of respondents: 10/219 = 5%). Two patients stated that they used a hormonal cream without further explanation of the usage (such as vaginal or nasal use). Only 7 patients stated that they had subcutaneous devices (proportion of respondents: 7/219 = 3%), 5 patients took hormonal vaginal rings (proportion of respondents: 5/219 = 2%) and 2 patients vaginal tablets (proportion of respondents: 2/219 = 1%). Hormonal IUDs were used in 20 patients (proportion of respondents: 20/219 = 9%) and non-hormonal intrauterine device (IUD) such as copper IUDs were used in only 8 patients (proportion of respondents: 8/219 = 4%; multiple answers were possible).

The main reasons for hormone intake were contraception (proportion of respondents: 162/222 = 73%), problems with their menstrual period (for example hypermenorrhea, dysmenorrhea or irregular menstrual cycle; proportion of respondents: 126/222 = 57%) and menopausal discomfort (proportion of respondents: 57/222 = 26%). Due to other diseases, 35 out of 222 women with HHT (= 16%), reported the intake of hormone preparations for medical reasons (endometriosis: 15/35 = 43%; osteoporosis: 9/35 = 26%; breast cancer: 5/35 = 14%; polycystic ovary syndrome: 5/35 = 14%; migraine: 1/35 = 3%). Almost every tenth female patient (proportion of respondents: 21/222 = 9%) stated that she took hormones because of skin and hair problems and 3 patients (3/222 = 1%) as fertility treatment (multiple answers were possible). A total of 21 patients reported on their experiences with estrogen and progestin combinations in relationship to epistaxis. Of those 21 patients 11 (52%) stated that they experienced an improvement and only 2 (10%) a worsening (Table 3).

**Table 3:** Influence of hormone intake on epistaxis and telangiectasia

**a)**	**Epistaxis** [N (%)]			
**Hormone intake**	**less**	**equal**	**more**	**Sum**
Estrogens only	1 (33)	2 (67)	0	3 (9)
Progestin only	2 (67)	1 (33)	0	3 (9)
Estrogen + progestin	11 (52)	8 (38)	2 (10)	21 (64)
Antiestrogen	1 (50)	1 (50)	0	2 (6)
Antiestrogen + estrogen	0	1 (100)	0	1 (3)
Antiestrogen + progestin	0	0	1(100)	1 (3)
Hormonal IUD	0	2 (100)	0	2 (6)
Sum	15 (45)	15 (45)	3 (9)	33
mean [95% CI], p-value	−0.36 [−0.60, −0.13], *p* = 0.003			

b)	Telangiectasia [N (%)]			
Hormone intake	less	equal	more	Sum
Estrogens only	0	1 (100)	0	1 (4)
Progestin only	1 (33)	0	2 (67)	3 (12)
Estrogen + progestin	2 (13)	12 (80)	1 (7)	15 (58)
Antiestrogen	1 (50)	1(50)	0	2 (8)
Antiestrogen + estrogen	0	1 (100)	0	1 (4)
Hormonal IUD	3 (75)	1 (25)	0	4 (15)
Sum	7 (27)	16 (62)	3 (12)	26
mean [95% CI], p-value	−0.15 [−0.40, 0.09], *p* = 0.212			

The table shows the patients who reported the active pharmaceutical ingredient. We asked for any hormonal preparation and contraception patients had ever taken in the last years. Thus, multiple answers were possible

There is a tendency toward fewer symptoms of hereditary hemorrhagic telangiectasia with the use of medication, which is significant in Table 3a (p-value = 0.003; less = −1, equal = 0 and more = + 1)

*IUD* intrauterine device, *N* number of patients, % percentage, *95%* *CI* 95 percent confidence interval

### Puberty, menstrual period and menopause in HHT

Most female patients stated that their epistaxis increased in puberty (more: 90 of 222 = 41%; equal: 75 of 222 = 34%; less: 57 of 222 = 26%) whereas most of them stated unchanged telangiectasia (more: 50 of 193 = 26%; less: 38 of 193 = 20%; equal: 105/193 = 54%).

**Fig. 2:**
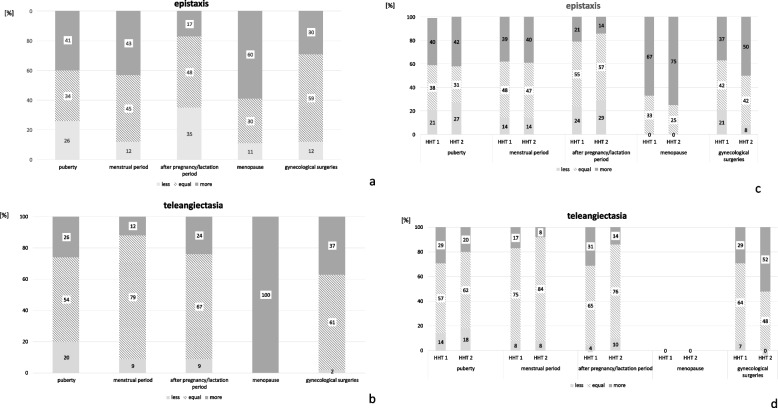
Effect of different hormonal status and gynecological surgeries on HHT symptoms. In their puberty, during their menstrual period and after their menopause most female patients with HHT noticed more epistaxis (**a** and **c**) and reported an increased formation of telangiectasia (**c** and **d**). After pregnancy/during lactation period more patients suffered from less than more epistaxis (**a** and **c**). HHT = hereditary hemorrhagic telangiectasia, HHT 1 = HHT type 1, HHT 2 = HHT type 2, % = data are shown in percentage; the number of respondents varied between 14 and 241

Many patients took hormones for treatment of hypermenorrhea (proportion of respondents: 54/222 = 24%) and dysmenorrhea (proportion of respondents: 40/222 = 18%). An irregular menstrual cycle as a reason for the intake of hormone preparations was reported in 14% (32/222). Before taking hormones, most women with HHT felt that they had a stronger menstrual bleeding compared to women without HHT at the same age (122/262 = 47%) with 43% (124/289) requiring four or more sanitary napkins or tampons per day. 69% of the patients (200/291) reported that on average their menstrual bleeding lasted for four to seven days. In 47 patients (proportion of respondents: 47/291 = 16%) a prolonged menstrual bleeding with more than seven days was documented. Regarding their menstrual period 25 women commented that they experienced more nosebleeds directly before the start of the bleeding (proportion of respondents: 25/95 = 26%). During their menstrual period and after their menopause most female patients with HHT noticed more epistaxis (Fig. 2a and c) and reported an increased formation of telangiectasia (Fig. 2c and d).

Regarding the HHT symptoms in these different hormonal situations of a woman’s life (puberty, menstrual period, after pregnancy/lactation period and menopause) no statistically significant differences between patients with HHT type 1 or 2 could be detected. However, during the lactation period there was a tendency that epistaxis and telangiectasia symptoms for patients with HHT type 1 worsened more than for patients with HHT type 2 (Table 4).

**Table 4:** Differences in means between HHT type 1 and 2 regarding various hormonal periods and HHT symptoms

**a)**		**Change in epistaxis**			
	**HHT 1**		**HHT 2**		**mean difference**
	N	mean [95% CI]	N	mean [95% CI]	mean [95% CI] p-value
Puberty	42	0.19 [−0.05, 0.43]	53	0.15 [−0.07, 0.38]	0.04 [−0.29, 0.37] *p* = 0.811
Menstruation	44	0.25 [0.04, 0.46]	58	0.26 [0.08, 0.44]	−0.01 [−0.28, 0.26] *p* = 0.950
Lactation	33	−0.03 [−0.27, 0.21]	42	−0.14 [−0.34, 0.06]	0.11 [−0.19, 0.42] *p* = 0.468
Menopause	3	0.67 [−0.77, 2.1]	8	0.75 [0.36, 1.14]	−0.08 [−0.83, 0.67] *p* = 0.808
Surgery	19	0.16 [−0.21, 0.53]	24	0.42 [0.14, 0.69]	−0.26 [−0.70, 0.18] *p* = 0.239

b)		Change in telangiectasia			
	HHT 1		HHT 2		mean difference
	N	mean [95% CI]	N	mean [95% CI]	mean [95% CI] p-value
Puberty	35	0.14 [−0.08, 0.37]	46	0.02 [−0.16, 0.2]	0.12 [−0.16, 0.40] *p* = 0.393
Menstruation	36	0.08 [−0.09, 0.25]	49	0 [−0.12, 0.12]	0.08 [−0.11, 0.28] *p* = 0.401
Lactation	26	0.27 [0.05, 0.48]	42	0.05 [−0.11, 0.2]	0.22 [−0.03, 0.47] *p* = 0.085
Surgery	14	0.21 [−0.12, 0.55]	21	0.52 [0.29, 0.76]	−0.31 [−0.69, 0.07] *p* = 0.106

Female patients with HHT responded to the question how their HHT symptoms (epistaxis (a) and telangiectasia (b)) changed during different time periods in life (worsened = + 1, equal = 0, improved = −1). Epistaxis and telangiectasia for patients with HHT type 2 tended to worsen more than for patients with HHT type 1 having had gynecological surgeries. Regarding the lactation period patients with HHT type 1 tended to show an increase in HHT symptoms. However, there were no significant changes between HHT type 1 and 2

The number of patients who answered the question about the telangiectatic lesion in their menopause was too low for statistical testing (N = 3; see also study limitations). HHT = hereditary hemorrhagic telangiectasia, HHT 1 = HHT type 1, HHT 2 = HHT type 2, N = number of patients, 95% CI = 95 percent confidence interval, surgery = gynecological surgery of the uterus, ovaries, or fallopian tubes

### Pregnancy in HHT

Most patients stated that they had been pregnant (proportion of respondents: 197/236 = 83%). One third of these women with HHT stated that their bleedings and the number of telangiectasia increased during pregnancy (proportion of respondents: 76/197 = 39%). Only 13% (25 of 197 respondents) experienced an improvement of their epistaxis and telangiectasia formation.

In another question, 52 female HHT patients reported in which gestational week (approximately) of their pregnancy these changes appeared. Most women experienced an increase of their HHT symptoms during their pregnancy. Especially in the second trimester, half of the patients noticed changes in their HHT symptoms (proportion of respondents: 26/52 = 50%) with 22 patients (proportion of respondents: 22/26 = 85%) suffering from more bleedings and telangiectasia. Regardless the trimester, most female patients reported a deterioration of their HHT symptoms (Table 5).

**Table 5:** Experienced changes of HHT symptoms in pregnant HHT patients

	**HHT symptoms** [N (%)]			
	**less**	**equal**	**more**	**Sum**
trimester 1	2 (15)	0	11 (85)	13 (25)
trimester 2	3 (12)	1 (4)	22 (85)	26 (50)
trimester 3	0	0	7 (100)	7 (13)
total pregnancy	1 (17)	0	5 (83)	6 (12)
Sum	6 (12)	1 (2)	45 (87)	52
mean [95% CI], p-value	0.75 [0.57, 0.93], *p* < 0.001			

Of the 46 patients who experienced changes during one of the three trimester 40 women stated increased HHT symptoms (87%). In addition, 5 women also reported more symptoms during their whole pregnancy

There is a significant trend for more symptoms during pregnancy (*p*-value < 0.001; less = −1, equal = 0 and more = + 1)

*HHT* hereditary hemorrhagic telangiectasia, *N* number of patients, *%* percentage of the HHT symptoms are calculated within terms

### Lactation period in HHT

During the lactation period 48% of the women (81 of 170 respondents) did not experience any changes, 35% (60 of 170 respondents) stated less and 17% (29 of 170 respondents) more epistaxis. Regarding their telangiectasia, 67% of the patients (106 of 158 respondents) did not experience any changes, 24% (38 of 158 respondents) suffered from enlarged telangiectasia and 9% had less telangiectasia (14 of 158 respondents, Fig. 2).

### Gynecological surgeries in HHT

Out of the 306 female respondents 122 (40%) stated that they underwent a gynecological surgery of the uterus, ovaries or fallopian tubes (any uterus surgery: cesarean section, complete/partial hysterectomy, endometrium ablation, curettage, removal of myoma/fibroid, conization, dissection of an arteriovenous malformation: 80 of 122 respondents = 66%; complete hysterectomy: 50 of 80 respondents = 63%; any ovarian surgery: 29 of 122 respondents = 24%; single sided ovariectomy: 10 of 29 respondents = 34%; both sided ovariectomy: 4/29 = 14%; ovary dissection without further information: 1 of 29 respondents = 3%; removal of an ovarian cyst: 14 of 29 respondents = 48%; surgery of the fallopian tubes: 9 of 122 respondents = 7%; multiple answers were possible). Regarding the effect of a bilateral ovariectomy, which includes the loss of hormone production in premenopausal women, there was no statistically significant difference on epistaxis or telangiectasia after the surgery (*p* > 0.05). In the questionnaire, however, there was no clear information about pre- or postmenopausal status at the time of ovariectomy. In benign lesions, ovariectomy is performed after natural menopause, therefore, it is plausible that an ovariectomy induces no further changes in hormone-dependent conditions. However, patients with HHT type 2 tended to show an increase in epistaxis and telangiectasia (Table 4).

## Discussion

### Effect of hormonal intake and gynecological surgeries on HHT symptoms

Estrogens and progestins are groups of sex hormones that play an important role in the developmental and reproductive phases of a woman’s life. The types and amounts of these hormones produced depend on a woman’s reproductive status. Their levels fluctuate throughout the reproductive cycle, from normal menstruation to pregnancy and eventually to the cessation of menstruation after menopause. Hormone production is highest during the reproductive years, beginning with menarche, and declines with increasing age, particularly during the peri- and postmenopausal periods [18, 19].

In our study, the primary indication for systemic estrogen and/or progestin use among female patients was contraception. About their epistaxis and telangiectasia, most patients reported either improvement or no change in symptoms. Only a small number of patients perceived disease progression associated with hormonal intake. Similarly, in patients with HHT, Harrison et al. reported an improvement in epistaxis with oral contraceptive use [20]. The rationale underlying this effect remains unclear. One hypothesis regarding the impact of daily intake of stable systemic estrogen concentrations on epistaxis is that estrogen may induce squamous metaplasia of the nasal mucosa, resulting in improved epithelial coverage and protection of the underlying vascular tissue, thereby reducing susceptibility to trauma [21]. However, the prothrombotic effects of hormonal agents, such as estrogen or progestin preparations, have also been proposed as contributing factors [22].

In addition, histological studies have demonstrated that estrogen increases the stability of the epithelium and the perivascular connective tissue [6, 23, 24]. Based on these observations, several small, non-randomized studies investigating systemic estrogen therapy reported a reduction in epistaxis in patients with HHT [25–28]. Furthermore, systemic progestin therapy was administered to three patients with notable clinical improvement [29]. Successful treatment of a male patient with oral synthetic androgen Danazol has also been reported [30]. Subsequently, Vase et al. conducted a randomized controlled trial in which 17 patients (females and males; age range in years [28–83]) received estrogen (estradiol valerate) and 14 received placebo for three months. The only statistically significant finding was a slight reduction in transferrin levels in the estrogen group [31]. To date, this remains the only randomized control trail evaluating systemic estrogen therapy in HHT and demonstrates only limited clinical benefit. Since then, the use of systemic estrogen therapy appears to have largely been abandoned. This may be attributed to two main factors. First, systemic estrogen was associated with adverse effects, particularly in male patients, including breast tenderness and enlargement, anorexia, fluid retention, flushing, loss of libido, and weight gain. Second, a direct association has been established between combined estrogen–progestin therapy and an increased risk of breast and gynecologic cancers, as well as thromboembolic events [32]. In addition, participants in the aforementioned studies received different estrogen formulations. Most contraceptives contain ethinylestradiol (EE), whereas hormone replacement therapy or estrogen monotherapy bases on estradiol or equine estrogens, which bind less strongly to estrogen receptors than EE. Consequently, differential effects of contraceptives and estrogen therapies beyond contraception can be expected.

With regard to local treatment options, topical estriol application has been suggested to have a beneficial effect on the severity of epistaxis in patients with HHT [33]. However, the only randomized, placebo-controlled trial evaluating topical nasal estriol therapy in patients with HHT failed to confirm this effect [34]. Notably, according to literature, locally applied estriol has no relevant systemic effects, which may explain the lack of efficacy of local treatment. Moreover, the absence of estrogen and progestin receptor expression in nasal telangiectasia in HHT has been reported [35]. In the present study, we assessed the use of systemic or vaginal hormone preparations but did not consistently specify local routes of administration in all cases (for example nasal or vaginal creams), which—particularly in the case of estriol – are not expected to influence HHT- related symptoms.

Selective estrogen receptor modulators (SERMs) have been proposed as an alternative therapeutic approach due to their more favorable risk profile. In a pilot study, Albiana et al. demonstrated that the molecular mechanisms of raloxifene involve counteracting the haploinsufficiency of ENG and ALK1, suggesting that raloxifene may represent a therapeutic option, particularly for postmenopausal women with HHT. Furthermore, in a double-blind, placebo-controlled study followed by a long-term clinical trial, Yaniv et al. showed that tamoxifen appeared to be an effective agent for the treatment of recurrent epistaxis [36–38]. In addition, SERMs are listed as a promising therapeutic option in the second international HHT guidelines [39]. In line with this, male patients in the present study also reported systemic hormonal intake for bleeding control. In vitro studies have shown that SERMs increase endoglin (ENG) and ALK1 expression at both the mRNA and protein levels, thereby improving endothelial cell function and potentially compensating for haploinsufficiency of these genes [36].

Regarding gynecological surgeries, no significant difference was observed between patients who underwent ovariectomy and those who had gynecological procedures not associated with hormonal changes.

### Effect of natural hormone status on HHT symptoms in women

In the majority of patients with HHT, first symptoms occur during puberty [40]. In our study, the mean age at disease onset was 18 years. Although female patients exhibited lower hemoglobin levels compared to men, in contrast to the general population, anemia occurred with similar frequency in both sexes among patients with HHT. Most female HHT patients reported a normal menstrual bleeding pattern and severity [41]. This suggests that recurrent bleeding from nasal or gastrointestinal mucosa has a greater impact on hemoglobin levels than menstrual blood loss. With increasing age, epistaxis severity increased in both sexes, possibly due to cumulative endonasal trauma over time [42]. However, the greater increase in epistaxis severity scores observed in female patients suggests that additional factors, such as hormonal status, may influence epistaxis severity. Around the age of 60 years, following menopause and the associated decline in estrogen levels, women appear to experience more severe epistaxis compared to men. Koch, Esther and Lewis (1952) observed in a small cohort of female patients with HHT that bleeding from telangiectasia worsened toward the end of the menstrual cycle and after radiotherapy-induced menopause – both periods characterized by low circulating estrogen levels.

Consistent with these observations, epistaxis has been reported to worsen with age in women, particularly after menopause, with more than 80% reporting increased severity after 45 years of age [12]. Our study yielded comparable results, with higher proportion of women reporting worsening epistaxis after menopause than during any other hormonal phase.

### HHT symptoms during pregnancy

Harrison et al. observed improvement in epistaxis associated with oral contraceptive use, as well as temporary improvement during pregnancy. This finding may be explained by increased estrogen levels during pregnancy, followed by withdrawal of estrogen after childbirth and placental expulsion, concomitant with a peaking in prolactin levels [20]. However, this effect was not evident in our study, as one third of women with prior pregnancies reported a worsening of HHT-related symptoms during pregnancy, while most women reported no change during the lactation period. With regard to pregnancy trimesters, most patients reported worsening HHT symptoms during the second trimester, a period characterized by marked increase in hormones such as estrogen and progestin. Effects like increase of blood volume and softening of connective tissue during this period might be relevant factors contributing to this observation. In line with our study results, Andorfer et al. also reported an increase in HHT symptoms in approximately one third of pregnant women [43].

### Study limitations

The questionnaire was administered online; therefore, we were unable to control whether it was completed in full or only partially. Thus, the number of respondents varied across questions, and some proportions were associated with wide 95% confidence intervals, limiting their generalizability of the findings. The survey comprised a large number of questions and required a considerable amount of time to complete. However, the reasons for missing data remain unknown. Patients who perceived changes in HHT symptoms during different hormonal phases may be overrepresented compared with those who did not experience any changes, potentially leading to an underestimation of patients reporting no change. The study was designed as a cross-sectional survey, a design that is inherently susceptible to the selection bias, information bias, and confounding.

To improve data accuracy, respondents were encouraged to consult their medical records when possible. As the questionnaire was available in two different languages, participants from German and English-speaking regions were likely overrepresented. Regardless of age, the survey was accessible to all patients with HHT; consequently, some participants were only able to complete sections relevant to their current life stage. In addition, because multiple responses were permitted for certain questions, a direct association between specific hormones or hormonal therapies and their effects on HHT-related symptoms could not always be established.

In the questionnaire, male respondents were instructed to skip questions related to hormonal status (questions 101–118 in Additional File 1), which may have distorted the proportion of male respondents for certain questions (e.g., hormone intake) that could also be applicable to men. Nevertheless, data from at least eight male patients (sex could not be determined in all cases) who responded to this section were included in the analysis.

With respect to patients’ sex, respondents were limited to binary options (male or female). As a result, data from individuals with discordance between genetic, gonadal, endocrinological, or psychological sex or gender were not captured.

Furthermore, questions addressing changes in epistaxis and telangiectasia during hormonal therapy and menopause were included only in the German version of the questionnaire and were not available in the English version.

The effect of hormonal changes on telangiectasia remains unclear. In our study, the development of telangiectasia across different hormonal phases was predominately reported as unchanged. It should be noted that these findings are based on subjective self-reporting; without objective measures to assess the number and size of telangiectasia, quantification of changes remains challenging. Moreover, the questionnaire was filled in several years after pregnancy and lactation (mean age of patients was 54 years), so there might be a recall bias.

## Conclusions

In our study, a potential beneficial effect of female sex hormones, such as estrogen or progestin, on the reduction of epistaxis was observed. This may partly explain why younger women appear to be less severely affected by HHT-related symptoms compared to men. With increasing age, however, declining levels of female sex hormones and progressive dryness of mucosal linings may contribute to increased epistaxis, as reported by female patients. Although the strength of evidence provided by our data is limited, the tentative off-label use of female sex hormones may represent a potential therapeutic option for selected patients with HHT after failure of established therapies and following careful individual risk–benefit assessment. Further studies are required to determine whether oral contraceptives themselves contribute to reduced symptom severity or whether the absence of daily hormonal fluctuations associated with standardized hormone intake accounts for the observed beneficial effects.

Contrary to previous study results, our data showed that one third of women perceived a worsening of HHT-related symptoms during pregnancy, particularly during the second trimester. The hypothetical mechanism is not fully understood: it is a period of few weeks characterized by drastic hormonal changes and sustained high estrogen and progesterone levels. Notably, our data did not reveal differences in symptom severity during menstrual bleeding, a period characterized by declining hormone levels. This suggests that long-term average hormone concentrations, rather than short-term hormonal fluctuations, may be more relevant to symptom severity. However, substantial inconsistencies between patient-reported outcomes and findings reported in the medical literature persist, indicating that additional, yet unidentified factors may play a role.

## Supplementary Information

Supplementary Material 1.

Supplementary Material 2.

## Data Availability

The datasets used and/or analyzed during the current study are available from the corresponding author on reasonable request.
