# Supplementary material for: Influence of hormonal status in hereditary hemorrhagic telangiectasia – analysis of an online patient questionnaire
Source: BMC Pregnancy Childbirth. 2026 Mar 20;26:388. doi: 10.1186/s12884-026-08928-2 (PMC13063478; doi:10.1186/s12884-026-08928-2)
Supplement: Supplementary file 2 — Supplementary Material 2. [file 12884_2026_8928_MOESM2_ESM.pdf]

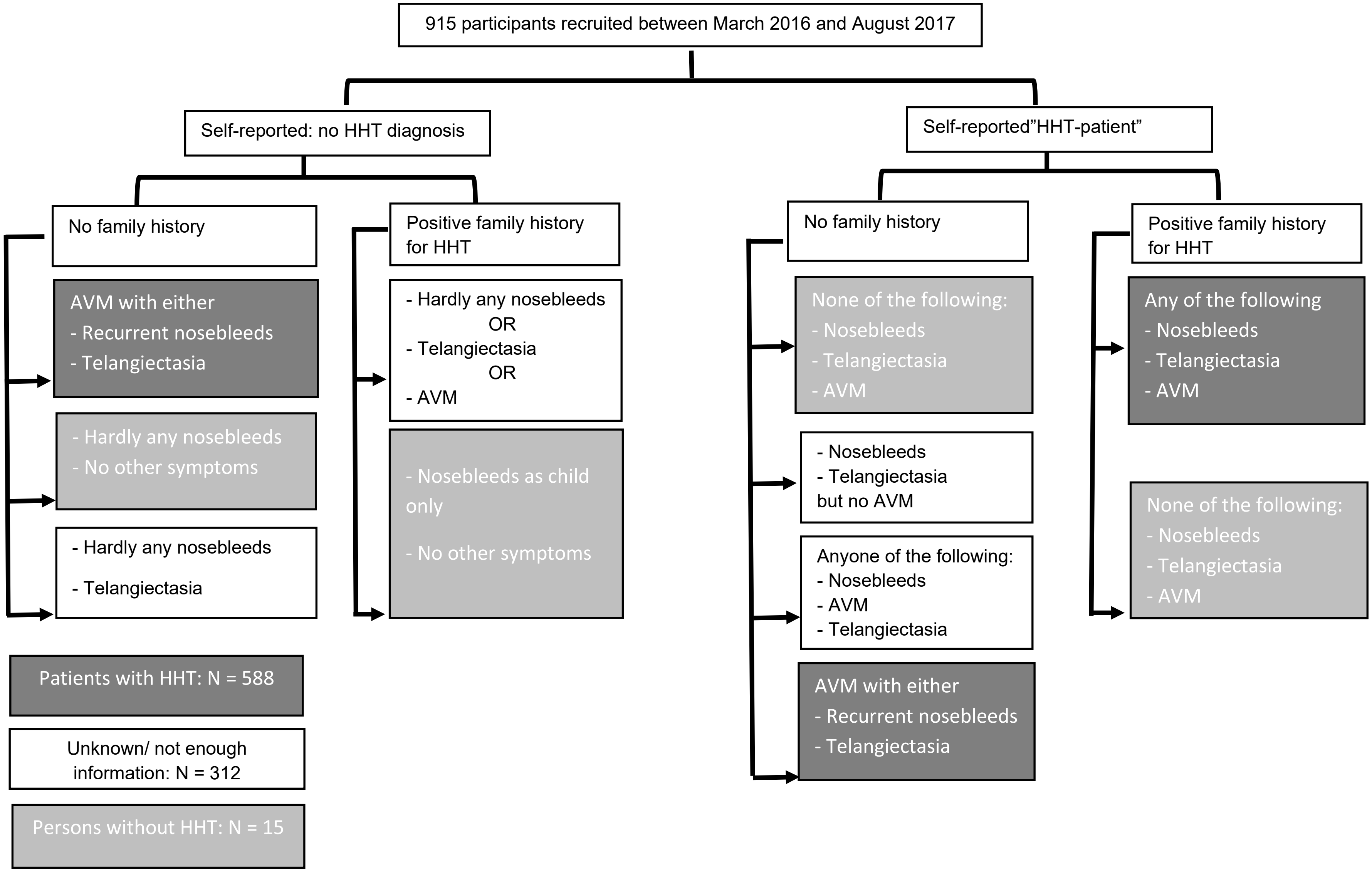

Stratification of diagnostic assignments

The diagnosis of HHT was established according to the previously published criteria [14]. It shows the modified Curaçao criteria. HHT = hereditary hemorrhagic telangiectasia, AVM = arteriovenous malformation (e.g. hepatic/ cerebral/ pulmonary vascular malformation or gastrointestinal involvement), N = number of participants.
